# Supplementary material for: Identification of photoperiod-induced specific miRNAs in the adrenal glands of Sunite sheep (Ovis aries)
Source: Front Vet Sci. 2022 Jul 22;9:888207. doi: 10.3389/fvets.2022.888207 (PMC9354845; doi:10.3389/fvets.2022.888207)
Supplement: Supplementary file 1 [file Data_Sheet_1.ZIP › Supplementary file/miRDeep2 file.zip!/miRDeep2 file/Know_Structure/SP42A1/oar-mir-3956.pdf]

miRBase precursor : oar-mir-3956
Total read count : 30
oar-miR-3956-5p read count : 30
oar-miR-3956-3p read count : 0
remaining reads : 0

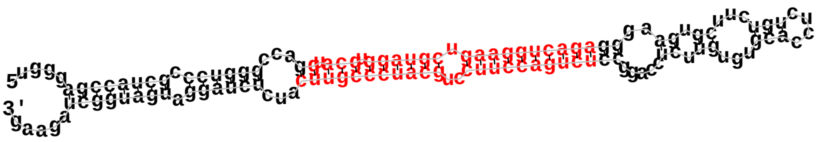

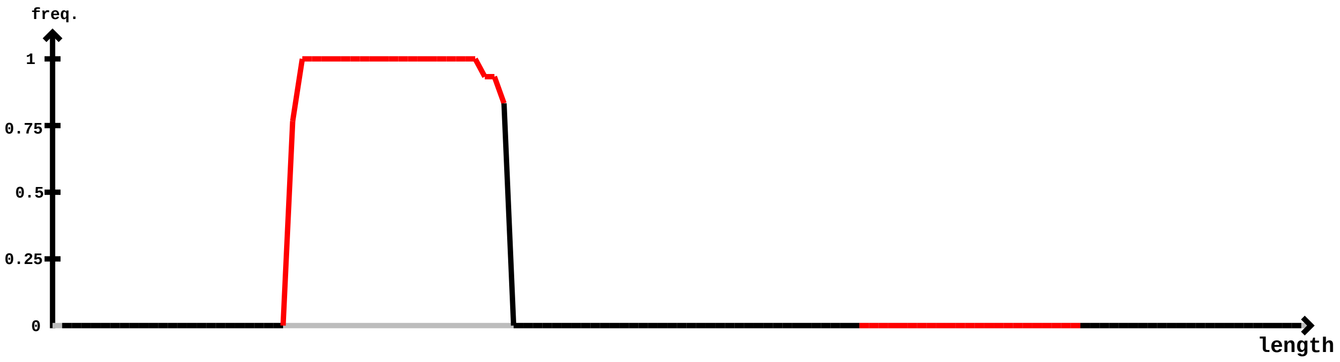


```
                                                                               oar-miR-3956-3p
                         oar-miR-3956-5p
5'- ugggagccaucgcccugggccagguacguggaugcugaaggucagagggaagugcuucugucuccacguguguucuccaggccucugaccuuccugcaucccguucaucucuaggaugauggcuagaag   -3'   exp
    ....(((((((.(((((...((.(((.((((((.(((((((((((..((.((...(((....)))...)).)).....)))))))))))..)))))))))))...)))))).)))))))).....   reads   mm
    .......................guacguggaugcugaagguc......................................................................................   2       0
    .......................guacguggaugcugaaggucag....................................................................................   3       0
    .......................guacguggaugcugaaggucaga...................................................................................   18      0
    ........................uacguggaugcugaaggucaga...................................................................................   7       0
```
